# Cardiac Autonomic Modulation - The Search for an Ultimate Technique

**DOI:** 10.5935/abc.20170166

**Published:** 2017-11

**Authors:** Esteban Rivarola, Mauricio Scanavacca

**Affiliations:** Instituto do Coração (InCor) do Hospital das Clínicas da Faculdade de Medicina da Universidade de São Paulo, São Paulo, SP - Brazil

**Keywords:** Bradycardia, Atrioventricular Block, Syncope, Vasovagal, Catheter Ablation, Ganglia, Autonomic

Abnormal parasympathetic activity on the heart has been implicated in several forms of symptomatic bradycardia, especially in young and otherwise healthy individuals. Neurally mediated syncope, sinus arrest and advanced atrioventricular (AV) block have high risk of injury and serious implications on quality of life. Nonetheless, until recently, their clinical management, mainly based on behavioral measures and pacing, have often proved to be ineffective or inadequate. An alternative approach to treat this specific population, avoiding device implants and continuous drug therapy seemed to be required.

Ganglionated plexus (GP) ablation was first described in 2005,^1^ with the purpose of targeting the main parasympathetic ganglia and promoting a vagal attenuation. During the last decade, several authors^2-8^ presented their clinical results, with significant remission of symptoms and electrocardiographic improvement, and autonomic modulation became an established therapeutic modality.

The cardiac autonomic nervous system, however, is not a simple target. Precisely the opposite: a complex interface between the central nervous system and the heart, comprising both extrinsic (Vagus nerve and sympathetic thoracic chain) and intrinsic components (epicardial GPs). It is widely recognized that most thoracic nerves and cardiac ganglia have both parasympathetic and sympathetic inputs (except the purely parasympathetic Vagus nerve and the purely sympathetic Stellate cardiac nerve), and present remarkable anatomic and functional heterogeneity. The variation and overlap of autonomic nervous supply to the myocardium makes interventional treatment difficult, although the predilection of certain structures for specific areas of the heart might be helpful for this purpose.

The intrinsic cardiac nervous system, a dense network of neuronal somata and connecting pre- and postganglionic fibers is the main target of the autonomic modulation procedures. Since almost all these epicardial ganglia have dual innervation, a titrated ablation with a net result of vagal attenuation must be reached.

Most authors considered as eligible for treatment those patients with no structural heart disease and recurrent functional bradycardia (cardioinhibitory syncope, advanced AV block or sinus arrest), after failure of medical treatment. Some authors^9^ recommend that a pre-ablation atropine test must be performed (0.04mg/Kg) and a positive response required as eligible criteria. Denervation has also been proposed to treat extreme bradycardia (pauses longer than 10 seconds) in asymptomatic patients,^7^ a very uncommon presentation with still unknown cardiovascular risks.

Several methods to identify the main GP implicated in functional bradycardia have been studied, alone and combined. Endocardial high-frequency stimulation has been widely used in previous articles,^2,5,6^ despite limitations caused by immediate rhythm disturbances and the requirement of specific equipment.

Spectral analysis of endocardial electrograms is an alternative method. Areas with right-shifted spectra (> 120 Hz) correlate with vagal-evoked response sites,^10^ and it has been used as a diagnostic parameter to identify GP sites.^1,3,7,9,11^

Due to technical limitations of above mentioned methods, the anatomic location alone, conducted based on previous anatomic studies^12,13^ emerged as a simple and attractive mapping strategy in recent articles.^6-8,14^ It is based on the concept that, although the structural organization of the intrinsic autonomic system varies from heart to heart, the most critical sites of innervation to the sinoatrial and AV nodes locate in predictable areas.

The cardiac autonomic nervous fibers spread through out the entire atrial epicardial surface; therefore a comprehensive ablation would be necessary to promote a significant atria autonomic modulation. However, most of autonomic ganglia are concentrated in some specific areas of the atria, allowing that a tailored amount of radiofrequency promote sufficient autonomic modulation and avoiding extensive lesions and potential complications. Recent clinical observations have contributed to our understanding of the sinoatrial node and AV node innervation^2,3,6,7,8,14,15^, and the interatrial septum emerged as a critical area, involved in most of the parasympathetic tone changes.^2,14^ Ablation of both sides of the septum, near the anterior right GP and inferior right GP of the left atrium, and the superior and inferior GP of right atrium had the greatest impact on heart rate and atrial-His interval.^14^ These data bring the perspective of performing ablation targeting the interatrial septum.

In specific cases, it was possible to observe that certain GP sites had differential effects on the sinoatrial and AV nodes, implying the presence of selective pathways in these structures.^8,14^ These data raised the possibility of treating sinus node arrest with selective denervation of the sinus node, and managing advanced AV block with selective AV node therapy, although we still lack clinical evidence that these patterns are consistent in large populations.

Amongst all the technical aspects of the autonomic denervation procedure, finding the best endpoints to measure vagal modulation is one of the most controversial issues in the field.^14-20^ Vagal-evoked response is a crude way to locate GP and, accordingly, evoked response abolition is a crude way to demonstrate clinical denervation. Spectral mapping with elimination of all right shifted signals can be useful but is a surrogate outcome and might result in large non-specific radiofrequency lesions.

Objective evaluation of the sinoatrial node and AV node function has been regarded as hard endpoints during denervation in recent articles.^3,8,14,21,22^ Authors observed a significant shortening of heart rate, AH interval and Wenckebach cycle length that, combined with a negative or significantly blunted response to atropine (0,04 mg/Kg) were considered procedural primary endpoints.

A recent work by Pachon et al.^11^ proposed a method of vagal stimulation by using an electrophysiological catheter placed in the internal jugular vein. This extracardiac technique has brought valuable data for denervation confirmation, although the presence of parasympathetic fibers also in most of the sympathetic thoracic nerves innervating the heart might limit its efficacy.^23^

Long-term follow-up results demonstrate that some technical aspects remain to be mastered, as symptom recurrence rates varying between 0 and 27%.^1,4,5,9,14^ Clinical limitations of the cardiac denervation procedure lie in the complexity of the intrinsic autonomic system: GPs behave as integration centers with extensive signal modulation that makes a uniform and permanently successful outcome unlikely. The highly dense neuroanatomy of the atria raises the possibility that a significant portion of the innervation remain stunned but still functional after ablation. In that case, a redo procedure might be helpful.

Late vagal tonus recovery is another important cause of recurrences. Metaiodobenzylguanidine imaging studies revealed that denervation persists for at least 4 months,^24^ but long-term evaluation are still lacking.

A large, multicenter clinical trial designed to determine the most proper method to perform denervation is of the essence. Yet, some available data seem consistent and valuable, as interventions on autonomic cardiac modulation became a worldwide standard procedure for management of functional bradycardia: 1- denervation is useful for refractory patients; 2- anatomic mapping emerge as a simple and effective approach; 3- the interatrial septum has a critical role, and 4- physiological evaluation (extracardiac stimulation, heart rate and AH interval shortening) combined with a negative atropine test seemed to be the most adequate endpoints.
